# Improving Awareness of and Engagement with Reflective Practice and Balint Groups Among International Psychiatry Doctors: A Pre-Post Webinar Survey Study

**DOI:** 10.1192/bjo.2026.11296

**Published:** 2026-06-30

**Authors:** Sanaa Moledina

**Affiliations:** Cambridgeshire and Peterborough NHS Foundation Trust, Cambridge, United Kingdom

## Abstract

**Aims:**

Reflective practice and Balint groups are essential for psychiatrists’ professional development, enhancing clinician wellbeing and job satisfaction. While embedded in UK psychiatric culture and training curricula, international doctors may have limited awareness of, exposure to, and access to these tools.

This study assessed international psychiatry resident doctors' baseline familiarity with and access to reflective practice and Balint groups, and evaluated changes in engagement following an educational webinar.

**Methods:**

Pre- and post-intervention surveys were distributed to international psychiatry doctors attending a webinar organized by the British Pakistani Psychiatrists’ Association (BPPA) on 22 November 2025. The pre-intervention survey assessed demographics, prior training in reflective practice, engagement frequency, workplace culture, and access to Balint groups. The post-intervention survey evaluated self-rated confidence and intended engagement in reflective practice. The intervention consisted of a one-hour webinar introducing reflective practice and Balint group principles, including a supervised Balint group demonstration led by a UK higher trainee.

**Results:**

The pre-session survey respondents (n=12) included 4 trainees and 8 non-training doctors, with 8 trained in Pakistan (6 practicing locally, 2 relocated to Ireland) and 4 from Malaysia, Botswana, Algeria, and Fiji. At baseline, most participants lacked formal training in reflective practice (83.3%) and access to Balint groups (91.7%), while 33.3% had no access to formal reflective platforms. Half perceived their workplace or training curriculum as placing low or no emphasis on reflection. Pre-session, 50% of attendees reported frequent engagement (often/always), primarily through supervisor meetings (58%), portfolio entries (25%), and team reflective meetings (25%), while two participants had never engaged in formal reflective practice.

Post-session, the participants reported high confidence in reflective practice (mean 4.25/5; 91.6% rated confidence ≥4/5), and 91.7% requested additional resources, reflecting heightened interest. Intended frequency of future engagement (often/always) increased to 75% post-session, compared with 50% reporting frequent engagement at baseline. Qualitative feedback demonstrated participants' intent to advocate for establishing local Balint groups and enthusiasm to integrate reflective practice into their daily clinical practice.

**Conclusion:**

Reflective practice is essential for professional development and wellbeing, particularly for early-career psychiatrists. This study demonstrated that international psychiatry doctors have limited understanding of and exposure to reflective practice and Balint groups, with minimal emphasis within workplaces or training programmes. A brief online educational session improved confidence and intended engagement in reflection, highlighting the value of international educational collaborations in upskilling psychiatrists globally.

